# Brain Mapping – Indications and Techniques by Alfredo Quinones-Hinojosa et al., (2019) 212 pp, 120 illustrations Hardback ISBN: 9781684200924 Thieme Publishers New York/Stuttgart

**DOI:** 10.1007/s00701-020-04312-x

**Published:** 2020-04-19

**Authors:** Sandro M. Krieg

**Affiliations:** Department of Neurosurgery, Klinikum rechts der Isar, Technische Universität München, Ismaninger Str. 22, 81675 Munich, Germany

After decades of clinical practice, brain mapping experienced an increased use over the last few years. Several studies not only proved its usefulness for safe resections; other studies also provided a high level of evidence on the oncological benefit of gross total resections for intrinsic brain tumors per se, thus requiring neurosurgeons to add mapping to their armamentarium.

Despite some literature on the topic, the currently published book edited by Quinones-Hinojosa et al. offers a comprehensive collection of information covering every aspect of neurosurgical brain mapping. Chapters include anatomy, preoperative modalities, general neurophysiology, anesthesiological considerations, etc. It continues with chapters on awake options for mapping various brain functions, such as insular mapping, ventral and dorsal stream, visual pathway, and pre-rolandic and Rolandic mapping. Apart from those topics, the book also covers techniques for asleep mapping of motor function, spinal cord, and brainstem. Very recent developments on postoperative approaches promoting functional recovery are also part of the collection as well as a chapter on radiotherapy of eloquent regions.

Figures and illustrations, in addition to references, were well-chosen by authors widely known in the field.

When it comes to particular details in depth, some other books might serve as an additional source; yet, this first edition hardback collection of 23 chapters and 196 pages offers all information necessary to start understanding, execute first steps, and bring your advanced experience to perfection when it comes to neurosurgical brain mapping. It is therefore a comprehensive aid for all levels of training for surgery of eloquent intrinsic brain tumors.

